# A Sensitive Aptasensor Using Biotin-Streptavidin System for Patulin Detection in Apple Juice

**DOI:** 10.3390/bios12020059

**Published:** 2022-01-23

**Authors:** Xiaoqian Tang, Qi Zhang, Maria Isabel Pividori, Zhaowei Zhang, Jean-Louis Marty, Gaëlle Catanante

**Affiliations:** 1 Oil Crops Research Institute, Chinese Academy of Agricultural Sciences, Wuhan 430062, China; wtxqtutu@163.com (X.T.); zhaowei_zhang@126.com (Z.Z.); 2 Key Laboratory of Detection for Mycotoxins, Ministry of Agriculture and Rural Affairs, Wuhan 430062, China; 3 Laboratory of Risk Assessment for Oilseeds Products, Ministry of Agriculture and Rural Affairs, Wuhan 430062, China; 4 Key Laboratory of Biology and Genetic Improvement of Oil Crops, Ministry of Agriculture and Rural Affairs, Wuhan 430062, China; 5 Quality Inspection and Test Center for Oilseeds Products, Ministry of Agriculture and Rural Affairs, Wuhan 430062, China; 6 Laboratoire BAE-LBBM USR 3579, Université de Perpignan Via Domitia, 52 Avenue Paul Alduy, CEDEX, 66860 Perpignan, France; 7 Grup de Sensors i Biosensors, Departament de Química, Universitat Autònoma de Barcelona, 08193 Bellaterra, Spain; Isabel.Pividori@uab.cat; 8 Sensbiotech, 21 Rue de Nogarede, 66400 Ceret, France; jlmarty@univ-perp.fr

**Keywords:** patulin, aptamer, lateral flow assay, on-site, apple juice

## Abstract

Patulin contamination in fruits, vegetables, and their products is considered a serious health risk factor for food safety and human health. Thus, a rapid, simple detection method for patulin is becoming important, which could provide a tool for routine screening and food surveys. The objective of this study was to develop a sensitive aptamer-based lateral flow assay (FLA) using Streptavidin functionalized gold nanoparticles for sensitive patulin detection. An excellent dynamic range for patulin detection was obtained (2.7~139.8 ng/mL in the buffer and 7.07~359.5 ng/mL in the sample) with no affinity for other mycotoxins such as zearalenone (ZEN), ochratoxin A (OTA), aflatoxin B_1_ (AFB_1_), citrinin or tenuazonic acid (TEA). The limit of detection was 0.19 ng/mL in the buffer and 0.36 ng/mL in the real sample. The recoveries were 83.3% to 107.1%, with a satisfactory RSD value from 6.5% to 7.5%. Hence the established LFA could be used as a rapid, simple, on-site screening tool for PAT determination in apple juice.

## 1. Introduction

Patulin (PAT) is a toxic secondary metabolite, naturally produced by several fungi species such as *Aspergillus*, *Penicillium*, and *Byssochlamys*. They typically grow on fruits, i.e., apples, pears, peaches, tomatoes, and grapes [1], and have also been reported in vegetables and cereal grains. PAT is found most often in rotting apples and products made from spoiled apples, and is frequently associated with the presence of *Penicillium expansum* [2]. Patulin was described initially as an antibiotic due to its strong activity against both gram-positive and gram-negative bacteria. Not long after, several studies suggested patulin was not only toxic to bacteria, but also toxic for animals and humans [3]. Ingestion of contaminated food with high levels of patulin has been shown to cause serious health issues in humans, especially in children. These acute symptoms include nausea, intestinal hemorrhage, convulsions, oedema, and agitation [4], and chronic exposure may lead to genotoxic, neurotoxic, and teratogenic effects [5]. However, strong evidence of its carcinogenicity in animals and humans is lacking. PAT was classified as group 3 by IARC (International Agency for Research on Cancer) [6].

Chemically, PAT (4 hydroxy 4H-furo [3, 2 c] pyran-2(6H)-one) is a heterocyclic lactone with a molecular weight of 154.12 g·mol^−1^ and low volatility. It is resistant to heat, between 105 and 125 °C, and is quite stable in aqueous medium, in the pH range between 3.5 and 5.5. These properties make it very difficult to eliminate through current food processing standards, even with pasteurization. Several publications and reports have shown levels of PAT contamination in apple products. In Europe, Torovic et al. [5] detected 21 positive samples of patulin contamination in 44 apple juice samples from Serbia destined for infant consumption, and 100% patulin positive apple juice samples destined for consumption by children. In addition, 42% positive results were detected in apple juice samples from the Czech Republic [6]; 30% positive samples from Tunisia [7]; 23.0% positive samples were detected in China [8]; 6.9% were detected in Japan [9] and 10% positive samples were detected in America [10]. Trade barriers usually result when to high levels of patulin in food exceeding the maximum limit allowable for importing and exporting countries. Patulin contamination poses a serious health risk, particularly to children who consume high levels of apple products during the 1st year of life (6.4 g.kg^−1^ body weight/d), compared with adults (1 g·kg^−1^ bw/d). This places children at increased risk for patulin toxicity. To limit public health risk, many regulatory agencies began placing caps on patulin levels destined for human consumption. The European Commission (EU) set the maximum concentration of 50 μg·kg^−1^ for patulin in fruit juices and 10 μg·kg^−1^ for baby foods [11]. While the U.S. Food and Drug Administration set a limit of 50 μg·kg^−1^ of patulin in fruit juices. 

This strict regulation has led to the development of faster and more specific and sensitive analytical methods with lower detection limits for patulin. Historically, PAT was identified using chromatographic techniques, such as thin-layer chromatography [12], high-performance liquid chromatography (HPLC) [13], gas chromatography (GC) coupled with UV detector [14], and chromatography tandem mass spectrometry (LC/MS) [15]. Despite their accuracy, these methods are expensive, time-consuming, and require qualified technicians [16]. Alternative methods such as bioassays and biosensors have received considerable attention since a polyclonal antibody against patulin was developed. Currently, several immunoassays have been developed using surface quartz crystal microbalance and plasmon resonance [17], near-infrared fluorescence [18], and chemiluminescence [19]. Additionally, cell [20], enzyme [21] and molecularly imprinted polymers (MIPs) are also being widely applied to develop biosensors [22]. All these methods provide highly sensitive assays for patulin identification. The strategies mentioned are advantageous due to their ability to signal amplify. However, several assays had issues with interfering effects [23], while some of the methods were costly and too complex to be performed in the field as they required technical expertise.

Lateral flow assays (LFAs) are representative on-site detection technologies and are based on a membrane containing detector and capture reagents. The method combines several benefits such as its user-friendly format, long-term stability, and cost-effectiveness. LFAs are widely used for qualitative and to some extent for quantitative monitoring, such as in food production or non-laboratory environments. 

Aptamers are single-stranded DNA or RNA that can specifically bind to a target molecule and offer significant advantages over antibodies as their production is less expensive and less labor-intensive^19^. Aptamers are easier to label with fluorescent dyes, enzymes, biotin, and DNA ligands, and they can be regenerated by heating. An added advantage of using aptamers is the option of hybridization with complementary DNA and the deconstruction of hybridization once aptamers bind with a target molecule. To date, there are only two publications addressing aptamers originally developed specifically for PAT. Wu et al. [24] reported PAT-11 exhibiting high affinity and excellent selectivity with a dissociation constant (K_d_) of 21.83 ± 5.022 nM, which were used for developing biosensors. Tomita et al. [25] reported an aptamer that could recognize PAT. Recently, studies on lateral flow technology based on aptamers were published for other types of mycotoxins. Shim et al. used fluorescence cy5 modified DNA probes as a direct signal amplification method to detect Aflatoxin B_1_ [26]. In addition, AuNPs-aptamer conjugates have been used for zearalenone [27] and ochratoxin A [28]. However, there have been no publications regarding aptamers based on LFA methods for patulin. 

We developed a competitive format aptamer-based lateral flow assay for patulin quantification. BIO-aptamer and DIG-DNA probes were used as recognition reagents. Anti-DIG and BIO-dendrimer were utilized as capture probes on the T line and C line, respectively. StreptAv-AuNPs, as a signal reporter, were used to capture both BIO-aptamer and BIO-dendrimer via streptavidin-biotin affinity. Based on our optimized parameters, we established an LFA for detecting patulin in apple juice.

## 2. Experimental 

### 2.1. Reagents, Materials, and Equipment

Patulin, polyclonal anti-Digoxin from sheep (anti-DIG), Tween-20, sucrose, and bovine serum albumin (BSA), were purchased from Sigma-Aldrich (Barcelona, Spain). InnovaCoat^®^ GOLD 40 nm; Streptavidin gold nanoparticles (streptAv-AuNPs) were purchased from Innova Biosciences (Cambridge, UK). Bio-dendrimers were generously provided by Prof. Isabel Pividori of Universitat Autònoma de Barcelona. Buffer solutions were prepared using Milli-Q water, and all other reagents were analytical reagent grade (supplied by Sigma-Aldrich and Merck). Buffer compositions were (a) Conjugate dilution buffer (2 mmol L^−1^ borate pH 7, 10% *w*/*v* sucrose); (b) sample pad buffer (0.01 mol L^−1^ phosphate buffer pH 7.4, 1% BSA, 0.05% Tween 20), and; (c) running buffer (0.01 mol L^−1^ phosphate buffer pH 7.4, 1% BSA, 0.05% Tween 20). Cellulose (111554) and glass fiber conjugate pads (491862) were purchased from Millipore (Billetica, MA, USA). Absorbent pads (1148506301) and nitrocellulose (NC) membranes (10547006) were purchased from GE Healthcare Life Sciences Whatman (Amersham, UK). To absorb the components onto the nitrocellulose strips, a Lateral Flow Reagent Dispenser from Claremont Bio (Upland, CA, USA) in combination with the KDS Legato™ 200 series syringe pump from KD Scientific Inc. (Holliston, MA, USA) was used to generate a straight and well-defined line. The intensity of the test and control bands were read using the ImageJ software.

### 2.2. Construction of the Lateral Flow Test Strips

First, the canal of the dispenser was cleaned using 5 mL of water, methanol, water, and air, respectively. An amount of 150 µL of the desired solution was placed in the dispenser at a rate of 38 µL·min^−1^. The solution was dispensed into the channel until it reached the end of the nitrocellulose membrane, forming a perfect, defined line. The dispenser was cleaned between each deposition. Using this method, the anti-DIG and BIO-dendrimer were spotted onto the NC membrane as T line and C line, respectively, and the strip was then left to dry for approximately 1 h.

Preparation of the conjugate pad: A final volume of 50 µL of streptAv-AuNPs (concentration ranging from 3.69 × 10^−12^ to 5.9 × 10^−11^ mol·L^−1^) was prepared by diluting commercially purchased Streptavidin gold nano-particles (streptAv-AuNPs) (10 OD, SPR peak 530 nm, molar extinction 8.42 × 10^9^ M^−1^·cm^−1^, molar concentration 1.18 × 10^−9^ mol·L^−1^) with the corresponding conjugate buffer as solvent (2 mmol·L^−1^ borate pH = 7, 10% *w*/*v* sucrose) and was then dispensed into the glass fiber conjugate pad. The pad was dried for approximately 2 h at room temperature. The strips were assembled on an adhesive backing card.

### 2.3. Aptamers and DNA Probes 

Biotin-modified aptamers to PAT and DIG-modified DNA probes with different lengths (13 mer and 10 mer) were purchased from Microsynth AG (Schutzenstrasse, Balgach, Switzerland).

- Biotin-modified aptamer (40 mer) sequence A: 5′-Biotin- GGC CCG CCA ACC CGC ATC ATC TAC ACT GAT ATT TTA CCT T-3′.
- DIG-modified DNA probes A (13 mer): -5′- DIG- AAG GTA AAA TAT C-3′.
- Biotin-modified aptamer (30 mer) sequence B: 5′- Biotin-CCT GCG GGC GCT GTT CGC CTA GTC GGA AGG-3′.
- DIG-modified DNA probes B (10 mer): -5′- DIG- CCT TCC GAC T-3′.

The sequences A and B in italic type represent the sequence of aptamers specific to PAT, derived from S, Wu et al. [24], and Y. Tomita et al. [25], respectively. Additionally, appropriate lengths of single-stranded DNA, complementary to the aptamer, were required to form a switching structure from aptamer/complementary DNA (a duplex DNA) to aptamer/target complex. Therefore, DNA probes A and B were designed for the development of the LFA. The sequences underlined on the DNA probes were portions that could hybridize with the aptamer.

Lyophilized oligonucleotides were dissolved with sterile and nuclease-free Milli-Q water to 100 μM and aliquoted and stored at −20 °C. These were then diluted to the desired concentration using binding buffer (0.1 M of PBS, 5 mM MgCl_2_, pH = 7.4), incubated at 90 °C for 5 min, and then cooled to 4 °C on ice for 5 min. Aptamer and probe solutions were incubated at RT for 20 min before use.

### 2.4. Developed Lateral Flow Test Strips for Patulin Detection

Once the strip is prepared, 100 μL of standard patulin solution or spiked patulin apple juice mixed with a defined concentration of aptamer or probe solution (1:1, *v*/*v*) were loaded onto the sample pad. Solutions flowed into the absorbent pad by capillary action. After a 5 min incubation, 100 μL of running buffer were loaded on the sample pad to remove unbound streptAv-AuNPs. After 10 min, images were captured for quantification of the optical signal on the T line. The resulting images were processed using ImageJ software (NHI) base on gray-scale value. To evaluate the performances of the test, a calibration curve was generated using apple juice spiked with patulin standard solution. A calibration curve was fitted and expressed as T/C versus the natural logarithm of the patulins concentration. 

### 2.5. Sample Preparation

Apple juice at 100% concentration was purchased from a supermarket in Barcelona, Spain. The juice was stored at 4 °C until analyzed. The pH of the 100% apple juice was adjusted from 3.3 to 7.0 using 2 M NaOH. To simplify the assay protocol, we compared three methods to prepare the apple juice to be analyzed. A: without any dilution; B: diluted 2 times with running buffer; C: diluted 4 times with running buffer. Samples A, B, C samples were run through a 0.22 μM filter before being analyzed.

## 3. Results and Discussion

### 3.1. Principle of Lateral Flow Strips 

**Figure 1:**
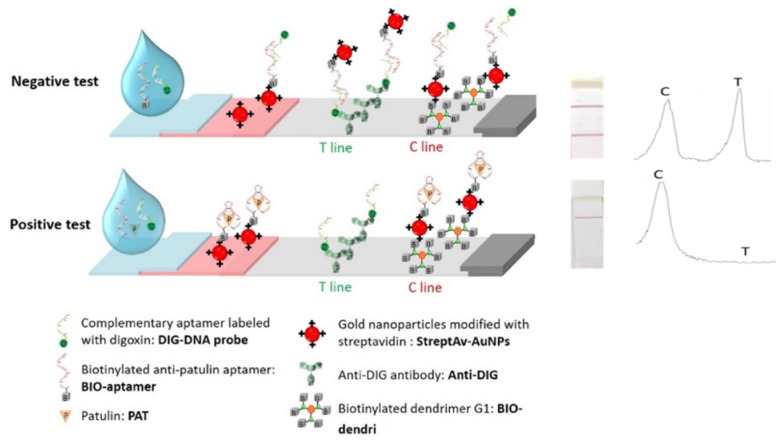
General scheme of strip design.

Figure 1 depicts the principle of the LFA for PAT detection. The format was based on a competitive test. Briefly, when the solution containing a mixture of DIG-DNA probe, BIO-aptamer, and PAT solution is dispensed onto the sample pad, PAT competes with DIG-DNA probe to bind the biotin-modified PAT specific aptamer. In the absence of patulin, the BIO-aptamer and DIG-DNA-probe completely hybridize to each other via double-stranded DNA complex formation (hybridized BIO-aptamer/DIG-DNA probe complex). The complex then migrates up to the conjugate pad and fixes onto streptAv-AuNPs. Subsequently, the streptAv-AuNPs/BIO-aptamer/DIG-DNA probe migrates to the test line to be captured by anti-DIG. Excess amounts of streptAv-AuNPs or streptAv-AuNPs-BIO-aptamers/DIG-DNA probe get trapped by the biotinylated dendrimers immobilized at the control line. Consequently, two red lines on the membrane are detected.

BIO-aptamers preferably bind with PAT, resulting in the inability to hybridize with the DIG-DNA probe. This, in turn, results in the absence of, or reduced, streptAv-AuNPs/BIO-aptamers/DIG DNA probe complexes available to be captured on the T line, resulting in reduced T line signal compared to the negative test without patulin.

### 3.2. Comparison and Selection of Aptamer Sequences and Competition Modes

Two aptamers were compared to obtain a more sensitive assay. *Sequence A* with a dissociation constant (K_d_) of 21.83 ± 5.022 nM (Kd) and *sequence B* with unterminated *Kd.* BIO-aptamer and DIG-DNA probe solutions (1:2, M/M) were incubated for 30 min. The binding ability of the aptamers was compared in the presence and absence of 100 ng/mL patulin. Results were evaluated by inhibition values [1]. In a competition mode, the lower signal in the presence of patulin, higher inhibition values were observed. This suggested the detection was much more sensitive. Inhibition values had a better sensitivity using sequence A (12.3%) compared to sequence B (5.2%). The inhibition values were calculated using the following equation:

(1) $$\%\;{\mathrm{Inhibition}}_{\mathrm{conc}.\mathrm{of}\;\mathrm{target}}=\left(1-\frac{{\mathrm{signal}}_{\mathrm{positive}\;\mathrm{test}}}{{\mathrm{signal}}_{\mathrm{negative}\;\mathrm{test}}}\right)\times 100$$

Based on the data, we selected sequence A to establish the patulin detection assay. The BIO-aptamer could bind patulin via multiple interactions or hybridize to the DIG-DNA probe. We asked how the complementary-aptamer used in the detection may affect the interaction between the BIO-aptamer and patulin. The specificity of the BIO-aptamer with DIG-aptamer is better compared to the target. Hence, we compared two different competition modes. One competition mode was to incubate DIG-aptamer, BIO-DNA probe, and patulin together in one step for 30 min. The second competition mode was to incubate the BIO-aptamer and sample solution together for 30 min, then added the DIG-DNA probe. Both competition modes worked with the following concentrations: 25 µg·mL^−1^ of anti-DIG, and 25 nM of BIO-aptamer and DIG-DNA probe. A signal decay on the T line was obtained when 100 µg·mL^−1^ patulin was analyzed. The inhibition value was 35.0% with the second competition mode compared to 29.9% for the first mode. Hence, we selected the second competition mode for developing the method.

### 3.3. Comparison of Detection Methods

We optimized the reaction time of the DIG-DNA probe and BIO-aptamer in the solution to increase the sensitivity of the assay. We coated the T line with anti-DIG (0.25 µg·mL^−1^) first for 20 min at RT, then coated with DIG-DNA probe (optimized concentration 12, 5 nM) overlapping with the anti-DIG location. We then incubated this for 1 h at RT to dry. The DIG-aptamers were then fixed on the T line location, which we recorded as method II (Figure S1). For method I, we only coated anti-DIG on the T line. In the presence of 100 µg·mL^−1^ of PAT, the inhibition was 59.1% with BIO-aptamer 25 nM (optimized concentration), while the inhibition for method II was 56.3%. The sensitivity of the two methods was similar. We decided on Method I because the protocol for preparing the T line of the strip was easier.

### 3.4. Optimization of BIO-Aptamer and DIG-DNA Probe Ratio

**Figure 2:**
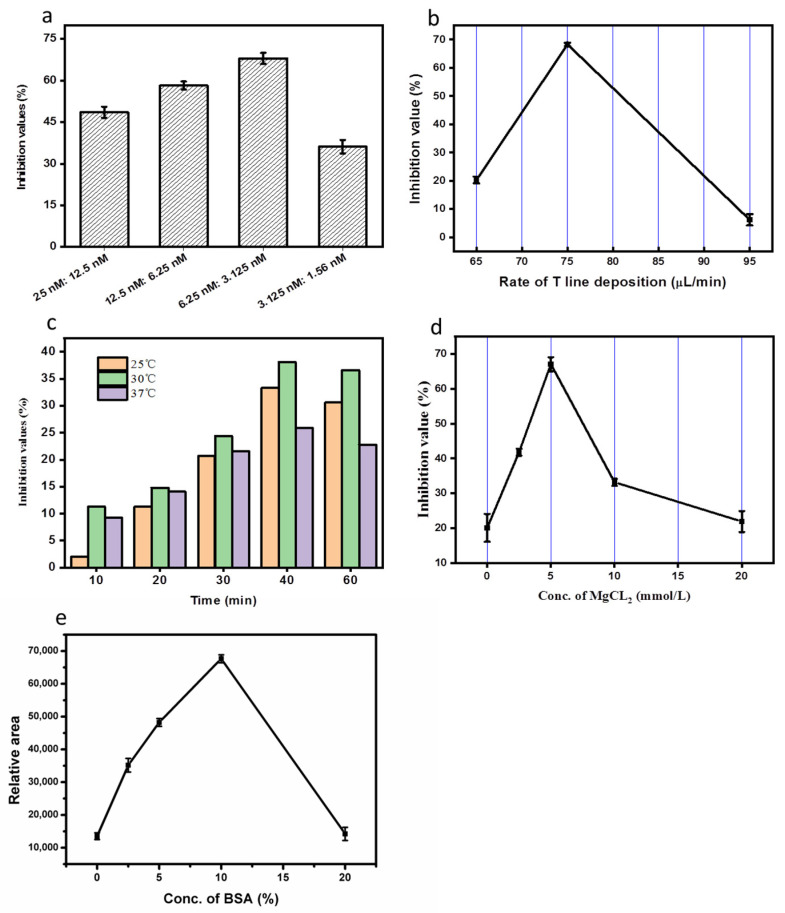
(**a**) Optimization of the BIO-aptamer and DIG-DNA probe concentration. A, 25.0:12.5; B, 12.5:6.25; C, 6.25:3.12; D, 3.12:1.56 nM; (**b**) Deposition rate of anti-DIG on the T line; (**c**) Incubation time and temperature of anti-DIG; (**d**) Concentration of MgCl_2_ in the running buffer; (**e**) Concentration of BSA in the running buffer.

In a competitive lateral flow assay, the sensitivity and the stability depend on the relative concentration of the aptamer. Our strategy was based on generating bridge-digoxin complementary aptamer hybrids with BIO-aptamers to determine a correlation between the signal reporter and anti-DIG antibody on the T line. Hence, the ratio and concentration of the aptamers primarily determined the sensitivity for patulin detection. We evaluated several molar ratios of BIO-aptamer/DIG- DNA probe (1:1, 1:2, 1:4, 1:6). The molar ratio of 1:1 exhibited the best inhibition percentage (13% with 100 ng/mL of patulin). However, we observed when the DIG-DNA probe ratio was increased, the sensitivity of the assay decreased (Table S1). We then reduced the molar ratio of the DIG-DNA probe (4:1, 3:1, and 2:1). The best results were obtained using a 2:1 ratio, with the concentration of the DIG-DNA probe being 2.5 nM. Furthermore, we optimized the assay using different aptamer concentrations (25.0:12.5, 12.5:6.25, 6.25:3.12, 3.12:1.56 nM), as shown in Figure 2a, where A, B, C, D represent the respective aptamer concentrations. In theory, the sensitivity should increase when aptamer concentration is reduced, however, at 3.12: 1.56 nM, the signal was too low to be quantified using the Image J software. We selected an aptamer concentration of 6.25:3.12 nM, which gave the highest inhibition signal of 68% in the presence of 100 µg·mL^−1^ patulin.

### 3.5. Optimization of Probe Concentration Immobilized on the Membrane

Parameters such as concentration of streptAv-AuNPs, anti-DIG antibodies; incubation time, and temperature were evaluated. We found that StreptAv-AuNPs used as the signal reporter had a negative correlation with patulin. Anti-DIG antibody was able to capture streptAv-AuNPs indirectly based on the bridge DIG-DNA probe/BIO-aptamer complex. We evaluated different dilutions of the streptAv-AuNPs commercial solution (25, 50, and 100 times) with 25 and 125 µg/mL of the anti-DIG antibody. The results showed that with 125 µg·mL^−1^ of the anti-DIG antibody, streptv-AuNPs must be diluted 10 times, otherwise the signal was not strong enough to be observed by the naked eye. When the anti-DIG antibody was increased to 250 µg/mL, the signal observed was too low using 100X dilution of streptv-AuNPs; with 25X dilution of streptv-AuNPs, the inhibition was 5.6%, and with less than 50× dilution of streptAv-AuNPs, the inhibition was 11.3%. Hence, 0.25 mg/mL of anti-DIG antibody and 50X dilution of streptv-AuNPs was selected as the optimized concentrations for the assay.

We then evaluated different speed rates of deposition of the anti-DIG antibodies onto the NC membrane (65 μL/min, 75 μL/min, and 95 μL/min). An amount of 75 µg·mL^−1^ showed the best inhibition of 68.3% with 100 ng/mL patulin and was higher compared to 95 and 65 µg·mL^−1^ (Figure 2b). In addition, we evaluated incubation times (10 min, 20 min, 30 min, 40 min) and incubation temperatures (25 °C, 30 °C, 37 °C) (Figure 2c). Based on the inhibition, we selected 30 °C and 40 min.

### 3.6. Optimization of BSA and Cation Concentration in the Running Buffer

Previous studies have reported that cations are essential for specific recognition of some aptamers [29]. We used different concentrations of MgCl_2_ in the running buffer to evaluate the binding capability of the aptamers. The concentrations were evaluated by testing the inhibition values in the presence and absence of 100 µg·mL^−1^ patulin. At concentration of Na^+^ at 0.1 M, we optimized the concentration of Mg^2+^ from 0 to 20 mM. We found that inhibition increased from 0 to 5 mM, then decreased from 5 to 20 mM, with a maximum inhibition around 5 mM of Mg^2+^. This was subsequently used in the running buffer (Figure 2d). 

BSA could be used to prevent the non-specific binding of the probe to the T line [26]. Different BSA concentrations (0, 2.5, 5, 10, 20%, *w*/*v*) were evaluated. At BSA concentrations of 0 to 10%, the signal was more intense with increasing concentrations of BSA, however, we observed at 20% BSA, the solution did not flow easily along the strip (Figure 2e). This demonstrated that BSA could affect the liquid chromatography rate to affect the reaction time of each of the recognition reagents. Finally, a 5% BSA solution was selected for the buffer solution to obtain good sensitivity and an appropriate level of signal to quantitate.

### 3.7. Specificity and Sensitivity of LFA for Patulin Detection

**Figure 3:**
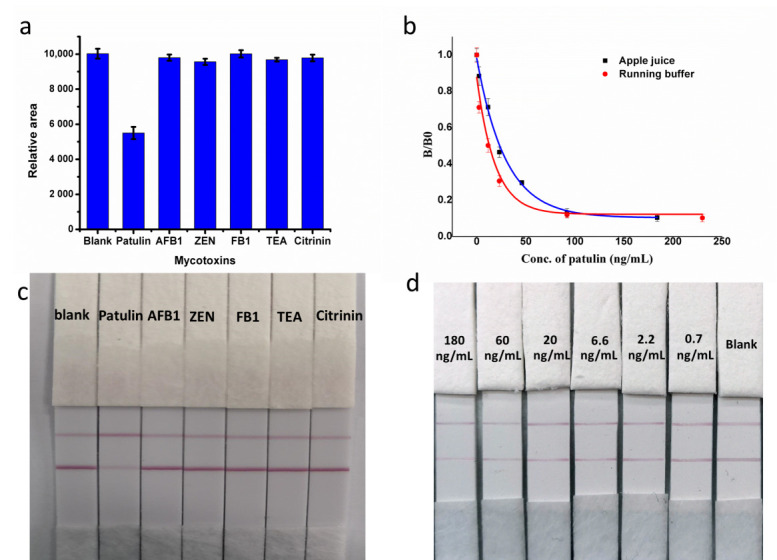
(**a**) Graph A represents the peak area obtained using Image software for images of LFA T line in the presence of patulin, AFB_1_, ZEN, FB_1_, TEA and citrinin; (**b**) Calibration curve and dynamic range of PAT for running buffer and apple juice samples; (**c**) Cross-reactivity test of the aptasenor with patulin, AFB_1_, ZEN, FB_1_, TEA and citrinin; (**d**) A series of patulin concentration (0–180 ng·mL^−1^) on aptasensor.

To evaluate the specificity of patulin LFA, the cross-reactivity with other mycotoxins, including AFB_1_, zearalenone (ZEN), and fumonisin B_1_ (FB_1_), citrinin and tenuazonic acid (TEA) were analyzed. An amount of 50 ng/mL patulin, and 500 ng/mL of AFB_1_, ZEN, FB_1_, citrinin TEA were spiked into the running buffer, respectively. The spiked standard solutions were added onto the sample pad and incubated for 10 min. A signal drop was observed on the LFA with patulin standard solution, while signals on the T line were unchanged compared to the blank for the other mycotoxins (Figure 3a,c). This suggested no cross-reactivity with other mycotoxins, demonstrating the patulin LFA had good specificity.

Sensitivity was evaluated by spiking running buffer with patulin standard solution. A standard curve was generated using the following equation, Adj.R-squar: 0.96434.

y = −0.03511 + 1.32328/1 + (x/51.18701)ˆ^0.5537^ (2)

The IC_20_ (the 20% inhibition concentration) value of the established LFA was 2.7 ng/mL, and the dynamic range was 2.7~139.8 ng/mL. The limit of detection (LOD) was 0.19 ng/mL, calculated based on a 3-fold SD of the T signal of the patulin-free buffer [30]. 

Sensitivity was evaluated by spiking sample extraction buffer with patulin standard solution. A standard curve was generated using the following equation, Adj.R-squar: 0.9969

y = 0.02779 + 0.97066/1 + (x/43.7611)ˆ^1.19702^

The IC_20_ value of the established LFA was 7.07 ng/mL, and the dynamic range was 7.07~359.5 ng/mL. The limit of detection (LOD) was 0.36 ng/mL, calculated based on a 3-fold SD of the T signal of the patulin-free sample (Figure 3b,d).

Several aptamer-based assays have been developed for patulin detection (Table 1). Assays based on fluorescent resonant energy transfer to measure patulin levels have been previously described by Wu et al. [31] and Ahmadi et al. [32]. These assays showed high sensitivity with LOD of 0.01 ng·mL^−1^ and 6 ng/mL, respectively. However, this is a complex assay that cannot be easily adapted for large-scale use. Enzyme-chromogenic [24] and electrochemical [33] aptamer-based assays have also been reported previously, with LODs of 0.048 ng·mL^−1^ and 2.8 ng·mL^−1^, respectively. However, the methods mentioned above were performed at a homogeneous phase. The advantage of our developed method was its wide sensing range with a LOQ that enabled the detection of patulin that satisfied the limits set by the European Commission. In addition, our method was a dipstick assay with a simple protocol that was easy to implement.

### 3.8. Validation of the LFA Method

The sensitivity of each sample was evaluated using the inhibition value of 100 ng·mL^−1^ patulin solution. We observed an inhibition of 37.2% using sample B (100% apple juice diluted 2 times with running buffer). The gold nanoparticles were tethered between the overlap of the conjugate pad and NC membrane when sample A (without any dilution) was used. Sample C (100% juice diluted 4 times with running buffer) presented an inhibition value of 34.8% and was similar to that of sample B. The highest dilution factor introduced large errors into the results. Based on these observations, we selected solution B. Patulin concentration, before and after filtration, was compared to evaluate the patulin loss during pretreatment. We spiked 5 concentrations of patulin and measured the concentration with filtration or without. A significant difference was observed when the calibration curves were compared. The recoveries from spiking with filtration were much lower compared to samples without filtration. Linear regression analysis produced a good correlation (y = 0.91x − 0.0747, R^2^ = 0.9746). Based on this, we calculated a loss of 9.07% of patulin when the sample pretreatment included filtration. 

To validate the analysis method, different patulin concentrations (10 ng·mL^−1^, 30 ng·mL^−1^, 60 ng·mL^−1^) were spiked with blank apple juice to determine recovery. The results were validated using HPLC. The recoveries using dipsticks were ranged from 83.3% to 107.1%, with a satisfactory RSD value from 6.5% to 7.5%, and had a good correlation with the HPLC results (Table 2). This provided confidence that the patulin dipstick method could be used to analyze actual samples. In addition, 10 natural samples with patulin contamination were measured using the LFA aptasensor and compared with high performance liquid chromatography (HPLC) (summarized in Table 3). A total of 5 samples were found to contain patulin with a concentration of lower than 2.6 ng·mL^−1^. The results showed satisfactory agreement between the aptasensor and HPLC, indicating that the LFA method was highly suitable for the screening and quantitation of patulin contamination in apple juice.

## 4. Conclusions

Contamination by mycotoxins in foods is becoming a global health problem. Identifying mycotoxin contamination as early as possible is essential for eliminating it from the food chain. Hence, it is important to screen samples using rapid, low-cost technologies that demonstrate high sensitivity and specificity. We developed an Aaptasensor assay based on ssDNA aptamers that could distinguish sample contamination using the naked eye. The dipstick assay is a technically easy method to implement and does not require complex coupling or synthesis of nanoparticle probes. The results could be read visually within 15 min, and the procedure could be performed easily by untrained personnel. In our study, we compared two different aptamers, competitive modes, detection methods, and optimization parameters which may contribute to the sensitivity of the assay. A calibration curve was established with a LOD of 0.19 ng·mL^−1^ and a quantitative range of 2.7~139.8 for the buffer and 7.07~359.5 ng/mL in the sample. The limit of detection was 0.19 ng/mL in the buffer and 0.36 ng/mL in the real sample. 

The recoveries for actual apple juice samples were from 83.3% to 107.1% and had a satisfactory RSD value of 6.5% to 7.5%. Although our LFA was not as sensitive as previously published complex methods used for analyzing homogeneous samples, it could be easily used during harvest, storage, and processing of food. The LOD for the method met the requirements for standard limits set by the EU for apple juice samples.

## Figures and Tables

**Table 1:** Published biosensors for the detection of patulin.

Method	Receptor	Signal Labled	LOD (ng/mL)	LOQ (ng/mL)	Dynamic Range	Ref.
Quartz crystal microbalance	antibody	/	0.022 ng/mL	/	Not calculate	[1]
Surface plasmon resonance	antibody	/	1.54 × 10^−5^ ng/mL	/	Not calculate	[12]
Conductometric	enzyme	/	50 ng/mL	/	Not calculate	[15]
Near-infrared fluorescence	antibody	/	0.06 ng/mL	/	Not calculate	[13]
Fluorescent resonant energy Transfor	aptamer	upconversion nanoparticles	0.003 ng/mL	0.01 ng/mL	0.01 ng/mL to 100 ng/mL	[29]
Colormetric	aptamer	/	0.048 ng/mL	0.05 ng/mL	0.050 to 2.5 ng/mL	[17]
Electrochemical	molecularly imprinted polymer	carbon dots	1.16 × 10^−7^ ng/mL	1.5× 10^−7^	1.5 × 10^−7^ to 1.5 × 10^−4^ ng/mL	[30]
Electrochemical	aptamer	ZnO nano flower	1.46 × 10^−5^ ng/mL	5 × 10^−5^	5 × 10^−5^ to 5 × 10^2^ ng/mL	[31]
Dipstick	aptamer	AuNPs		2.7 ng/mL	2.7 to 139.8 ng/mL	our work

**Table 2:** Recovery percentages obtained using the patulin dipstick for apple juice.

Spiked Concentration (ng/mL)	LFA Method			HPLC Method		
	Mean(ng/mL)	Recovery (%)	RSD (%)	Mean (ng/mL)	Recovery (%)	RSD (%)
10.0	10.71	107.1	7.5	11.53	115.3	4.2
30.0	24.99	83.3	6.5	22.20	74.0	3.9
60.0	59.20	98.6	6.8	58.54	97.6	4.7

**Table 3:** Validation of LFA using HPLC for patulin contamination in actual samples.

Sample	LFA Method (n = 5)		HPLC Method (n = 5)	
	Mean (ng/mL)	RSD (%)	Mean (ng/mL)	RSD (%)
1	ND ^a^	/	ND	/
2	3.58	6	3.53	4.5
3	4.28	7.2	4.20	5.5
4	ND	/	ND	/
5	ND	/	ND	/
6	2.93	6.8	2.86	6.7
7	ND	/	ND	/
8	5.38	6.5	5.56	5.8
9	2.89	7.8	2.65	4.2
10	ND	/	ND	/

^a^ ND: not detected.

## Supplementary Materials

The following supporting information can be downloaded at: https://www.mdpi.com/article/10.3390/bios12020059/s1, Details of buffers and solutions; optimization of the different ratios of BIO-aptamer and DIG-aptamer, schematic of the Method II, the structures of aptamer-40 and aptamer-3 are provided in one table and two figures. Table S1. Optimize different ratio of BIO-aptamer and DIG-aptamer; Figure S1. Schematic of the Method II, the different with Method I was the DIG-aptamer was fixed on the T line, according to the anti-DIG antibody; Figure S2. The structure of aptamer-40 and aptamer-30.
