# Supplementary material for: A Sensitive Aptasensor Using Biotin-Streptavidin System for Patulin Detection in Apple Juice
Source: Biosensors (Basel). 2022 Jan 23;12(2):59. doi: 10.3390/bios12020059 (PMC8869234; doi:10.3390/bios12020059)
Supplement: Supplementary file 1 [file biosensors-12-00059-s001.zip › biosensors-1516629-supplementary.pdf]

biosensors

Supplementary Information

A Sensitive Aptasensor Using Biotin-Streptavidin System for Patulin Detection in Apple Juice

Xiaoqian Tang [1,2,3,4,5,6], Qi Zhang [1,2,3,4,5,*], Maria Isabel Pividori [7], Zhaowei Zhang [1,2,3], Jean-Louis Marty [8] and Gaëlle Catanante [6,*]

[1] Oil Crops Research Institute, Chinese Academy of Agricultural Sciences, 430062 Wuhan, China; wtxqtutu@163.com (X.T.); zhaowei_zhang@126.com (Z.Z.)
[2] Key Laboratory of Detection for Mycotoxins, Ministry of Agriculture and Rural Affairs, 430062 Wuhan, China
[3] Laboratory of Risk Assessment for Oilseeds Products, Ministry of Agriculture and Rural Affairs, 430062 Wuhan, China
[4] Key Laboratory of Biology and Genetic Improvement of Oil Crops, Ministry of Agriculture and Rural Affairs, 430062 Wuhan, China
[5] Quality Inspection and Test Center for Oilseeds Products, Ministry of Agriculture and Rural Affairs, 430062 Wuhan, China
[6] Laboratoire BAE-LBBM USR 3579, Université de Perpignan Via Domitia, 52 Avenue Paul Alduy, CEDEX, 66860 Perpignan, France
[7] Grup de Sensors i Biosensors, Departament de Química, Universitat Autònoma de Barcelona, 08193 Bellaterra, Spain; Isabel.Pividori@uab.cat
[8] Sensbiotech, 21 rue de Nogarede, 66400 Ceret, France; jlmarty@univ-perp.fr
* Correspondence: zhangqi@oilcrops.cn(Q.Z.); gaelle.catanante@univ-perp.fr (G.C.)

Buffers and solutions:

The composition of different buffer were prepared as the follows:

Coating buffer: 0.01 mol/L phosphate buffer saline (PBS), pH 7.4; supplemental with 0.5 % w/v BSA

Conjugate diluting buffer: 0.002 mol/L borate pH 7.0; supplemental with 10% w/v sucrose.

Binding buffer: 0.1 mol/L phosphate buffer, pH 7.4, supplemental with 0.005 mol/L $MgCL_2$.

Running buffer: 0.1 mol/L binding buffer, pH 7.4; supplemental with 5% BSA (w/v), 0.05 % Tween 20 (v/v)

Table S1. Optimize different ratio of BIO-aptamer and DIG-aptamer.

Ratio (mol:mol)	BIO-Aptamer (μmol/L)	DIG-Aptamer (μmol/L)	Inhibition Value (%)
1:1	0.025	0.025	13
1:2	0.025	0.050	12.3
1:4	0.025	0.125	None
1:6	0.025	0.150	None

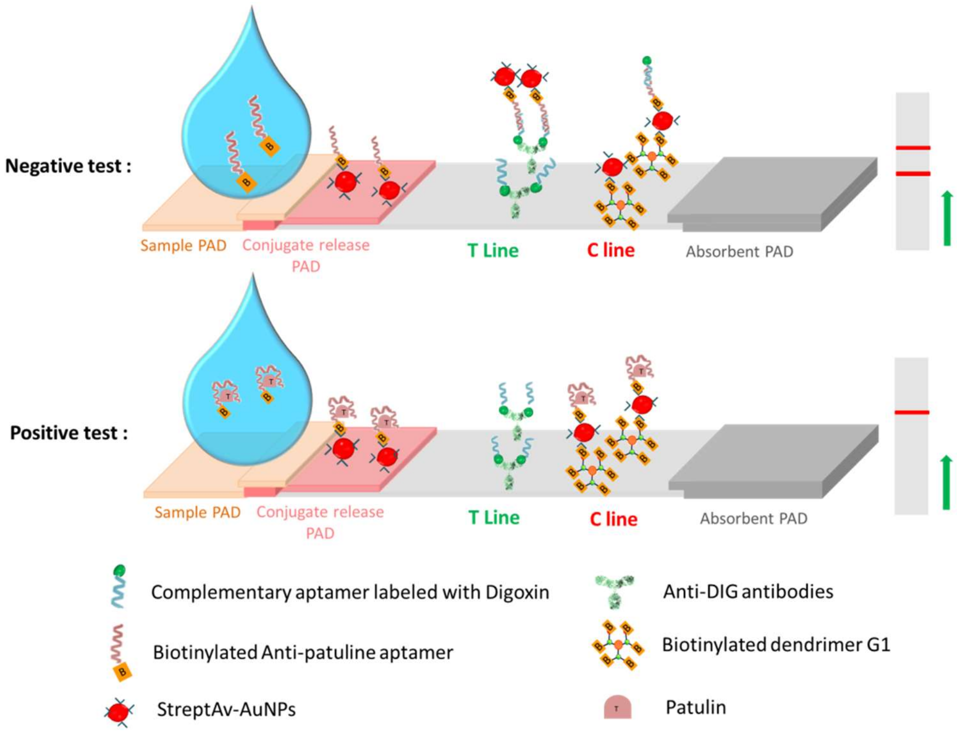

Figure S1. Schematic of the Method II, the different with Method I was the DIG-aptamer was fixed on the T line according the anti-DIG antibody.

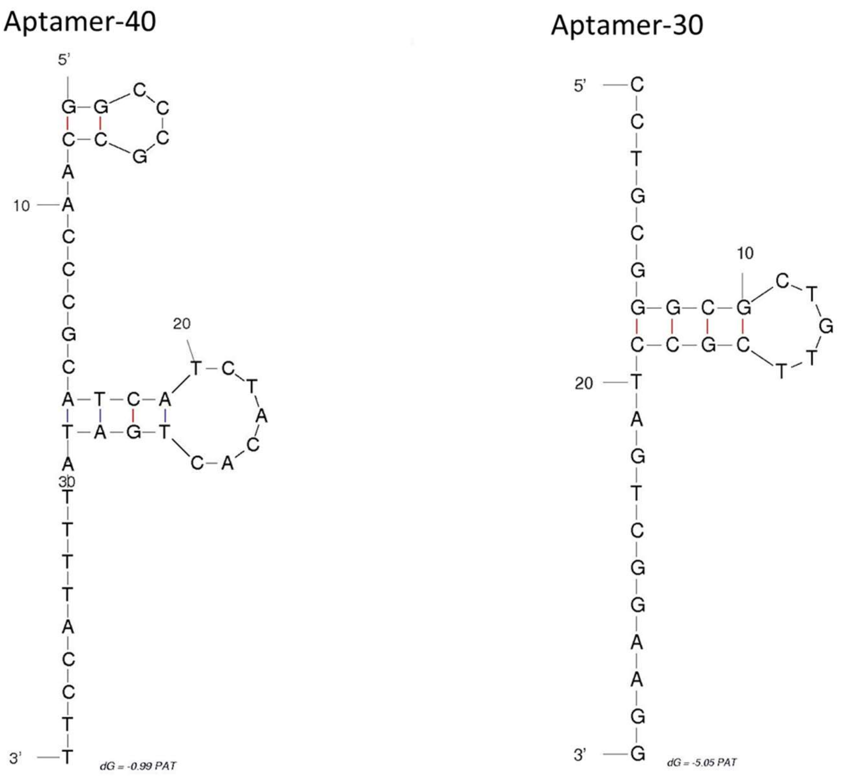

Figure S2. The structure of aptamer-40 and aptamer-30.
